# Preparation and Characterization of New Electrospun Poly(lactic acid) Nanofiber Antioxidative Active Packaging Films Containing MCM-41 Mesoporous Molecular Sieve Loaded with Phloridzin and Their Application in Strawberry Packaging

**DOI:** 10.3390/nano12071229

**Published:** 2022-04-06

**Authors:** Yuan Xie, Guiguang Cheng, Zhoushan Wu, Shang Shi, Jinghao Zhao, Lin Jiang, Dengbang Jiang, Mingwei Yuan, Yudan Wang, Minglong Yuan

**Affiliations:** 1 School of Chemistry and Environment, National and Local Joint Engineering Research Center for Green Preparation Technology of Biobased Materials, Yunnan Minzu University, Kunming 650500, China; yuan18787028972@sina.com (Y.X.); jianglin@ymu.edu.cn (L.J.); 041814@ymu.edu.cn (D.J.); 041808@ymu.edu.cn (M.Y.); 2 Faculty of Food Science and Engineering, Kunming University of Science and Technology, Kunming 650500, China; ggcheng@kust.edu.cn; 3 Key Laboratory of Chemistry in Ethnic Medicinal Resources, State Ethnic Affairs Commission & Ministry of Education, Yunnan Minzu University, Kunming 650500, China; 21227038010037@ymu.edu.cn (Z.W.); 15807220798@sina.cn (S.S.); jinghaozhao1019@sina.com (J.Z.)

**Keywords:** phloridzin, MCM-41, electrostatic spinning, antioxidant activity, antibacterial activity, active food packaging

## Abstract

Health concerns about food safety have increased in recent years. In order to ensure the safety and increase the shelf-life of food, many methods have been used to slow down the oxidation rate of food fat. In order to solve this problem, a new type of antioxidant-active packaging has emerged. Poly(lactic acid) (PLA) films containing phloridzin adsorbed on to an MCM-41 mesoporous molecular sieve were prepared by electrostatic spinning, using PLA as a film-forming substrate, phloridzin as an antioxidant, and MCM-41 as the adsorption and controlled release carrier. The physical properties of the new films—including microscopic structure, water vapor transmission rate, and fresh-keeping effects, as well as the mechanical, thermal, antioxidant, and antibacterial properties—were studied. When the mass ratio of MCM-41 to phloridzin is 1:2, the nanofiber membrane achieves a 53.61% free-radical scavenging rate and better antibacterial performance (85.22%) due to the high content of phloridzin (30.54%). Additionally, when the mass ratio of the molecular sieve to phloridzin is 1:2 and 3:4 (with the best antibacterial performance of 89.30%), the films significantly delay lipid oxidation in the strawberry packaging, allowing the fresh-keeping time to be extended to up to 21 days before mildew appears. In this study, an MCM-41 mesoporous molecular sieve was used to load phloridzin for the first time. The packaging film with phloridzin, MCM-41, and poly(lactic acid) were used as the raw materials and electrospinning technology was used to prepare the packaging film with antioxidant activity. The packaging film was used for the first time in the packaging of strawberries.

## 1. Introduction

In recent years, due to the increasing health concerns about food safety and the widespread environmental pollution caused by non-biodegradable food packaging, the development of biopolymer membranes has attracted increasing attention [1,2,3,4,5]. Films used for food packaging should protect food from external contaminants and prevent chemical, physical, and biological changes during storage [6,7]. Conventional packaging provides several food protection functions and plays a role in blocking oxygen and moisture [8,9]. Recent developments in materials science and bioengineering research have focused on a new packaging technology that can improve quality and food safety [10,11]. Active packaging allows the active compound to diffuse into the food environment continuously and for an extended length of time, thus reducing the need for preservatives [12,13,14,15]. The development of bio-based polymer films has attracted increasing attention because of the growing focus on food health and safety, as well as the extensive environmental pollution caused by the non-biodegradability of existing food packaging [16]. In the last decade, based on the development of controlled release technology and the antioxidant requirements of fatty foods, antioxidant-active food packaging based on controlled release has also become a frontier hotspot in the field of active food packaging research [17,18]. Research developments on packaging film substrates and antioxidants, new controlled release technologies, and controlled release mechanisms have received extensive attention from researchers in related fields.

Phloridzin is a dihydrochalcone glucoside [19,20] with many important biological functions such as decreasing blood sugar [21], improving memory, anti-oxidation, and combating cancer. It has broad application prospects in the development of new drugs and natural health foods. For example, studies have shown that apple tree branches, leaves, and bark are rich in phloridzin.

PLA is a biodegradable and environmentally friendly material with the advantages of safety, great mechanical properties, high utilization rate, and low cost [22,23]. In recent years, PLA has been widely used in the food industry in food packaging due to its good tensile strength and ductility. 

First reported to be synthesized by scientists from Mobil Corporation in 1992, MCM-41 is a mesoporous molecular sieve material with a large pore volume and a uniformly distributed pore size that is adjustable within a certain range. It is widely used in the adsorption of harmful substances in the environmental field and the study of catalysts [24,25,26]. In 2001, in the drug research field, Vallet et al. [27] first used MCM-41 as an adsorbent to adsorb ibuprofen molecules for drug molecule delivery. Subsequently, MCM-41 has received extensive attention from researchers as a controlled-release carrier for drugs. However, in the field of active packaging, the use of MCM-41 as a controlled-release carrier loaded with phloridzin is still relatively low.

Thus, this work aims to develop a novel antioxidant-active packaging film containing phloridzin adsorbed onto an MCM-41 mesoporous silica sieve by electrostatic spinning. The film was selected based on the mass ratio of MCM-41 to phloridzin to finely control the release of the antioxidant from the packaging materials. Moreover, the physical and active properties of the new film were investigated to analyze the changes that may be caused by the addition of different amounts of the phloridzin/MCM-41 assembly. Finally, the performance of the film was studied through the fresh-keeping time of strawberries [28,29].

## 2. Experimental Section

### 2.1. Main Materials and Reagents

Anhydrous ethanol (chemically pure) and 95% ethanol (chemically pure) were acquired from Sinopharm Chemical Reagent Co., Ltd (Shanghai, China). Dichloromethane (DCM) (molecular formula CHCCl_2_) (analytical purity) was acquired from Tianjin Damao Chemical Reagent Factory (Tianjin, China). Ultra-pure water was processed in the laboratory. Phloridzin was isolated with the purity of >98% in the laboratory. Hexadecyltrimethylammonium bromide (chemically pure), concentrated sulfuric acid (chemically pure), and sodium silicate nonahydrate (chemically pure) were acquired from Sinopharm Chemical Reagent Co., Ltd (Shanghai, China). Poly(lactic acid) (PLA), molecular formula (C_3_H_4_O_2_)n, was self-made in the laboratory. N, N-Dimethylformamide (DMF) (molecular formula (CH_3_)_2_NOCH) was acquired from Tianjin Komiou Chemical Reagent Co., Ltd. (Tianjin, China), and DPPH was acquired from Sigma-Aldrich (Shanghai, China) Trading Co., Ltd.

### 2.2. Main Experimental Instruments and Equipment

The main equipment and instruments required for the experiment, their models, and their manufacturers, are presented in Table 1.

### 2.3. Experimental Method

#### 2.3.1. Synthesis of MCM-41 Mesoporous Molecular Sieve

A typical synthesis of MCM-41 by hydrothermal method was conducted as follows. Sodium metasilicate nonahydrate (25 g) was dissolved in 30 mL of de-ionized water. CTMABr (6.4 g) was heated in 20 mL of de-ionized water. They were then mixed by vigorously stirring for 10 min. After being cooled to room temperature, the pH value was adjusted to 10 with 5 mol/L H_2_SO_4_. Subsequently, the mixture was transferred into a Teflon-lined stainless-steel autoclave and kept at 130 °C for 72 h. Finally, the mixture was washed with de-ionized water until the pH value reached 7. It was then dried overnight at 90 °C and calcined at 550 °C for 5 h to obtain a sample of MCM-41 mesoporous molecular sieve.

#### 2.3.2. Phloridzin on MCM-41 Mesoporous Molecular Sieve

Phloridzin was dissolved in a small amount of dimethyl sulfoxide (DMSO). Then, a specific amount of MCM-41 powder and dichloromethane (DCM) were added. After magnetically stirring at room temperature for a certain amount of time, to obtain the phloridzin/MCM-41 intermediate, the dichloromethane was removed by ultrasonic heating and drying. The loading rate of phloridzin in the assembly determines the antioxidant content in the active film with the same assembly amount, and the antioxidant content is directly related to the antioxidant capacity of the active film. Therefore, the orthogonal test method was used to study the effects of three factors in the preparation process of phloridzin/MCM-41 assembling on the loading rate of phloridzin. In the study, three levels were set for each factor, and an orthogonal experiment scheme was designed (Table 2).

#### 2.3.3. Electrospinning Preparation of PLA Antioxidant-Active Packaging Film

Solution preparation: The amount of PLA particles was weighed to 7 wt.% of the spinning solution, then a solution was added with a DCM/DMF ratio of 5:1. After magnetically stirring for 10 h, different ratios of phloridzin/MCM-41 intermediate powder were mixed to obtain a spinning solution. The antioxidant film was prepared using the electrospinning method. The spinning process parameters were set as follows: the receiving distance was 10–13 cm; the spinning voltage was 9–15 kV; the spinning temperature was 25 °C; the relative humidity was 65%; and the spinning speed of the main micro-push pump was 0.002–0.0030 mm/s. The obtained fiber film was dried at 45 °C for 8 h and sealed for subsequent experiments [30,31,32].

### 2.4. Testing and Characterization

#### 2.4.1. Thermogravimetric-Differential Scanning Calorimetry

Phloridzin, MCM-41, and the phloridzin/MCM-41 assembly were analyzed via thermogravimetric-differential scanning calorimetry using NETZSCH STA-449 F3 (Bavaria, Germany). About 3 mg of the mixture was weighed into the crucible and heated from room temperature to 950 °C in a nitrogen atmosphere. The heating rate was maintained at 10 °C/min with a flow rate of 25 mL/min. 

#### 2.4.2. Analysis of Pore Structure Parameters

The BET (Brunauer–Emmet–Teller) surface area, pore volume, and average pore diameter of the samples as prepared were obtained via nitrogen physisorption in ASAP 2420 equipment (Micromeritics, Norcross, GA, USA). The MCM-41 and phloridzin/MCM-41 assembly were separately degassed under vacuum at 100 °C for 6 h, and then 40 °C for 24 h, prior to the measurement.

#### 2.4.3. Infrared Characterization

The infrared analysis of the samples was conducted with Bruker TENSOR 27 Fourier Transform Infrared Spectrometer (Karlsruhe, Germany), with a test range of 4000–500 cm^−1^. The powder sample was processed by the potassium bromide tableting method. About 200 mg of potassium bromide (KBr) powder and 2 mg of the powder sample were mixed in an agate mortar until fully ground. The samples were pressed into self-supporting wafers and subsequently dried under an infrared lamp for testing. 

#### 2.4.4. X-ray Diffraction (XRD) Analysis

Powder X-ray diffraction (XRD) patterns were conducted using a Bruker D8 Advance X-ray laboratory diffractometer (Karlsruhe, Germany) with Cu Ka radiation (40 kV, 40 mA). A step scan mode was used with a scan rate of 0.01°/min (2θ) per second from 1.8° to 8°.

#### 2.4.5. Microscopic Morphology of the Film

Scanning electron microscope (SEM) measurements were performed with a NOVA NANOSEM-450 (Hillsboro, OR, USA) scanning electron microscope operating at 300 kV. The samples were removed from the aluminum foil, pretreated in a vacuum drying oven at 45 °C for 2 h, and sprayed gold for testing.

#### 2.4.6. Determination of Antioxidant activity

ABTS radical scavenging activity was determined according to the following procedure. To dissolve the phloridzin and the poly(lactic acid) in the film, 0.1 mL each of dimethyl sulfoxide (DMSO) and dichloromethane (DCM) were added. The solution was diluted with water until the concentration of the solvent in the solution was 1 mg/mL. The sample solution of different concentrations was mixed with 4 mL of ABTS (7 mol/L). After 6 min of reaction, absorbance was recorded using a Spectra Max M5 microplate reader (Sunnyvale, CA, USA). All the tests were performed in triplicate. The antioxidant activity of each sample was expressed as 50% inhibitory concentration (IC_50_), which indicated that the concentration of the sample had a 50% ABTS radical scavenging effect. [33]. 

#### 2.4.7. Determination of the Antibacterial Properties of the Film

A count of the flat colonies was used to evaluate the antibacterial performance of the fiber membrane sample, and the reported data corresponded to the average value of three similar runs. The agar diffusion method was used for the antibacterial evaluations. The antibacterial activity tests for the synthesized nanocomposites were conducted with the bacterial strain *E. coli*. The ability of different samples to destroy *E. coli* was certified based on the decrease in number of the colonies observed on the agar plates [34]. The fiber membrane inhibition rate, R, was calculated according to Equation (1):

(1) $$\;R=\frac{A-B}{A}\times 100\%\;$$

where R is the bacteriostatic rate (%); A is the average number of colonies in the PLA fiber membrane sample without phloridzin in the control group; and B is the average number of colonies in the PLA fiber membrane sample with the phloridzin/MCM-41 assembly added.

#### 2.4.8. Research on the Mechanical Properties of the Film

The tensile strength and elongation at break of the film were assessed using a computer-controlled electronic universal testing machine (CMT4104). Before stretching, the samples were pretreated for at least 40 h at 23 ± 2 °C and 50 ± 10% relative humidity. Each sample was cut into a rectangle of similar size, and its width was measured. The tests were performed on rectangular film specimens (10 mm × 90 mm) until fracture with a uniform crosshead speed of 1 mm/min. At least 5 specimens were assessed for each sample.

#### 2.4.9. Water Vapor Transmission Rate of the Film (WVP)

A beaker containing 12 g of anhydrous silica gel was sealed with the manufactured film and then placed in a desiccator with a saturated sodium chloride solution at the bottom. The water vapor transmission rate (WVP) of the film was measured by the needle method. It was initially weighed once every 1 h, and then once every 12 h after stabilization [35,36].

The WVTR and the WVP were calculated according to Equations (2) and (3):

(2) $$\mathrm{WVP}=\frac{\mathrm{WVTR}\times C}{∆P}$$

(3) $$\mathrm{WVTR}=\frac{W}{t\times S}$$

where WVTR is the water vapor transfer rate, g mm/m^2^·d·kPa; ∆P is water vapor pressure difference; C is film thickness, mm; W is water increase, g; t is time, h; and S is the area of the membrane, m^2^. The saturated vapor pressure of pure water at 25 °C was 3.1671 kPa.

#### 2.4.10. The Fresh-Keeping Effect of Antioxidant Film on Strawberry

All the fruits were sorted, and were selected with the following criteria: same size, color, and maturity, no pests, and no mechanical damage. The strawberries were evenly divided into 11 groups. The strawberries were put into matching prepared beakers that had been cleaned and dried. The beakers were then sealed with films of different compositions, and blank beakers without films were denoted as KB. During the test, samples were taken once a day and two indicators were used for evaluation: sensory indicators and weight loss rate, sensory indicators are shown in Table 3. Each evaluation was repeated three times, and the results were averaged [37,38,39].

The weight loss rate was calculated according to Equation (4)

(4) $$W=\frac{m_{1}-m_{2}}{m_{1}}\times 100\%$$

where W is the weight loss rate, %; *m*_1_ is the mass of the strawberry before storage, g; and *m*_2_ is the mass of the strawberry after storage, g.

## 3. Results and Discussion

### 3.1. Thermogravimetric Analysis

**Figure 1:**
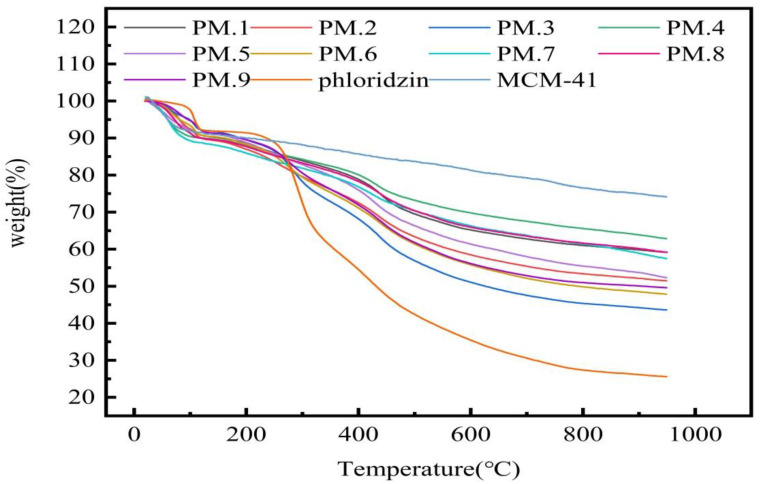
TGA curves of phloridzin/MCM-41 assemblies.

The TGA curves of phloridzin, MCM-41, and the phloridzin/MCM-41 assembly are shown in Figure 1. The assembly was prepared under the conditions that test NO. 1 was recorded as PM.1, and so on. Accordingly, in the TGA curves of MCM-41 and the phloridzin/MCM-41 assembly, slight weight loss was detected when the temperature was raised from 35 °C to about 100 °C, corresponding to the water loss [40]. At about 950 °C, the weight loss of the sample was minimal, indicating that most of the phloridzin had decomposed. In the process of heating up to 950 °C, the weight loss of phloridzin/MCM-41 was divided into three parts: the weight loss of the water absorbed by MCM-41 in the sample; the weight loss of the pyrolysis of phloridzin; the weight loss due to pyrolysis of phloridzin. However, it can be seen from the phloridzin curve that the weight loss of phloridzin was basically unchanged at around 950 °C, so the weight loss of phloridzin is negligible [41]. The loading rate _AT_ of phloridzin in the phloridzin/MCM-41 assembly is shown in Equation (5):

(5) $${\;\mathrm{Loading}\;\mathrm{rate}}_{\mathrm{AT}}=W_{M}-W_{\frac{\mathrm{AT}}{M}}$$

where, W_M_ and W_AT/M_ represent the weight residue rate (%) of MCM-41 and phloridzin/MCM-41 assembly at 950 °C, respectively. They can be directly measured with a thermogravimetric analyzer.

The calculated loading rate of phloridzin in the phloridzin/MCM-41 assembly is shown in Table 4. Sample number PPM.3 reached the highest loading rate of phloridzin in the assembly (30.54%), and had a mass ratio of molecular sieve to phloridzin of 1:2, with a stirring time of 16 h. Sample number PPM.4 had the lowest phloridzin loading rate (11.29%), and had a mass ratio of molecular sieve to phloridzin of 3:1, with a stirring time of 8 h. The difference in loading rates between the two samples was 19.25%.

As shown in Table 5, the three factors were arranged in descending order. The largest range of the three factors was Factor I. Factor II and Factor III were positively correlated with the load factor. It was concluded that during the preparation of the phloridzin/MCM-41 assembly, the proportion of phloridzin had the greatest influence on the loading rate of phloridzin in the assembly and was, therefore, the decisive factor among the three. The content of phloridzin increased with the increase in loading rate, while the content of MCM-41 and the mixing time had less impact on the loading rate. Among them, when the content of MCM-41 was the lowest, the sum of the load factors was maximized. 

### 3.2. Analysis of the Pore Structure of MCM-41 before and after Loading

**Figure 2:**
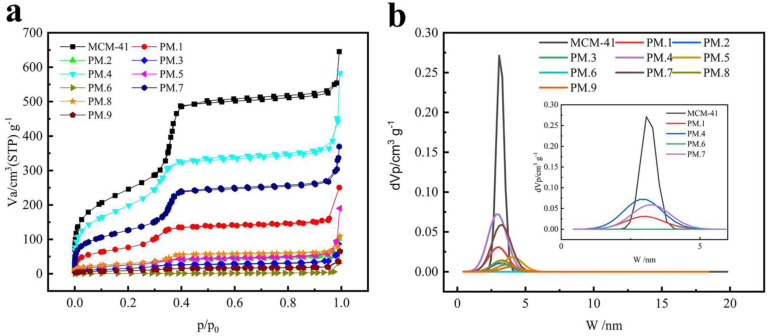
(**a**) N_2_ adsorption/desorption isotherms and (**b**) pore size distribution of pure MCM-41 and phloridzin/MCM-41 assembly.

The nitrogen adsorption/desorption isotherms and the pore size distribution, respectively, of MCM-41 and phloridzin/MCM-41 are shown in Figure 2a,b. As shown in Figure 2a, MCM-41 demonstrated typical irreversible type IV adsorption isotherms. In addition, N_2_ molecules were adsorbed on MCM-41 in the first stage of the monolayer adsorption process. Capillary condensation occurred when the relative pressure (P/P_0_) rose over 0.35. The adsorption of MCM-41 with phloridzin had type I isotherm with a type H4 hysteresis loop at a high relative pressure. These characteristics may be related to the presence of micropores and the absorption of molecules into pores that are about the same diameter as the adsorbate molecule.

The N_2_ adsorption capacity of the phloridzin/MCM-41 assembly was significantly lower compared with that of the pure MCM-41 sample, indicating that the phloridzin was loaded into and occupied the pore space of MCM-41. In addition, different from the pure MCM-41, the loaded MCM-41 sample exhibited hysteresis in the low-pressure zone where the relative pressure was lower than 0.35. This means that micropores appeared in the sample and that the adsorbate entered the pore channel. In other words, phloridzin entered the pores of MCM-41 and was loaded successfully.

As shown in Figure 2b, the pore size distribution of MCM-41 was relatively concentrated before and after loading. The pore size distribution of the phloridzin/MCM-41 assembly shows that the pore size decreased after adsorption, implying that the antioxidant had been successfully loaded into the inner channels of the mesoporous matrix. In addition, the reduction of the surface area and pore volume from 890.740 to 59.504 m^2^·g^−1^, and from 0.961 to 0.133 cm^3^·g^−1^, respectively, after adsorption, proves the successful loading of phloridzin by MCM-41.

The data reveal that the specific surface area and pore volume of the assembly gradually decrease with the increase of the load rate, as shown in Table 6.

### 3.3. XRD Analysis before and after MCM-41 Loading

**Figure 3:**
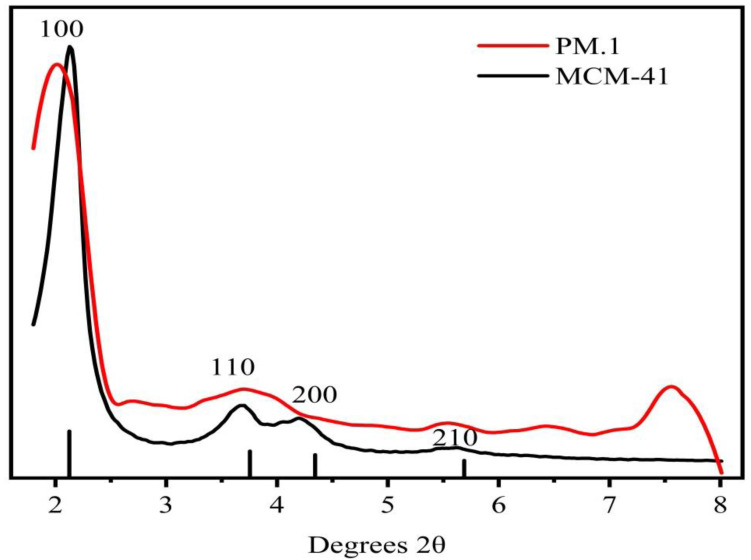
Small-angle XRD diffraction patterns of MCM-41 and assembly sample 1.

As can be seen in Figure 3, all the materials retained the crystal structure of MCM-41. The peaks at 2–3° and 3–6° correspond to (100), (110), (200), and (210) crystal surfaces. The 2*θ* angles correspond to different positions of the characteristic peaks, indicating that only the interplanar spacing of the molecular sieves are different. The composition of the molecular sieves did not change.

Small-angle XRD patterns of two samples are depicted in Figure 3. The characteristic peaks of the MCM-41 molecular sieve in the figure are consistent with the XRD pattern of MCM-41 molecular sieve in the original literature. The presence of (100), (110), (200), and (210) crystal planes in the structure indicates that the sample also had the representative structure of MCM-41. [42]. Additionally, several characteristic peaks in the assembly samples were still relatively obvious. Therefore, the loading of phloridzin did not completely damage the ordered structure of MCM-41, and the molecular sieve was retained [25]. 

### 3.4. Analysis of the Microscopic Morphology of the Film

**Figure 4:**
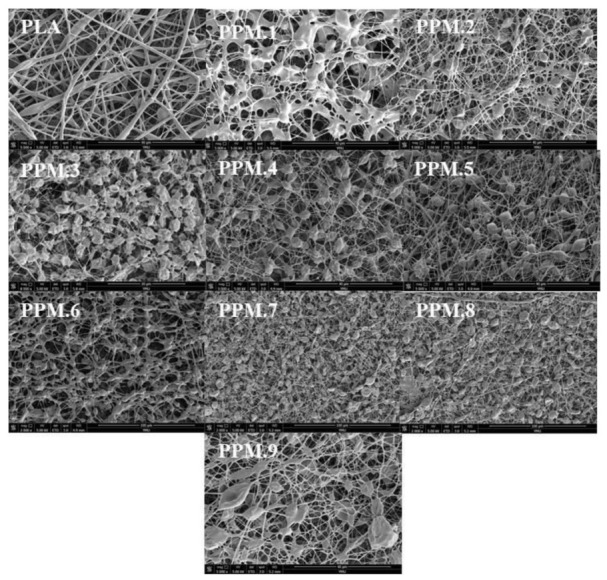
SEM micrographs of pure PLA and PLA films loaded with different amounts of phloridzin/MCM-41 assembly.

The anti-oxidation film prepared by electrospinning was obtained according to the sample number of the assembly according to the different films added: film sample 1 was recorded as PPM.1, and so on. The microscopic morphologies of the films with different components are shown in Figure 4. The effect of the assembly on the size and distribution of nanofibers in the antioxidant-active membrane is shown in Figure S1 of the Supplementary Materials.

It can be seen from Figure 4 that the uniformity of the PLA film gradually deteriorates with the increase in the assembly amount added. The pure PLA membrane has a uniform texture, and the amount of molecular sieve in the assembly remains unchanged. With the addition of phloridzin, the uniform structure of the membrane gradually deteriorates. With the addition of the molecular sieve and the active substance phloridzin added to the assembly, the uniformity of the antioxidant film becomes worse, resulting in the uneven distribution of fibers on the film. The phloridzin/MCM-41 assembly added to the film is visible as the nodule-like structures in Figure 4.

### 3.5. Infrared Spectrum Analysis of the Film

The labeling of the infrared characteristic peaks of the assembly and the active film infrared is shown in Figures S2 and S3 in the Supplementary Materials. 

**Figure 5:**
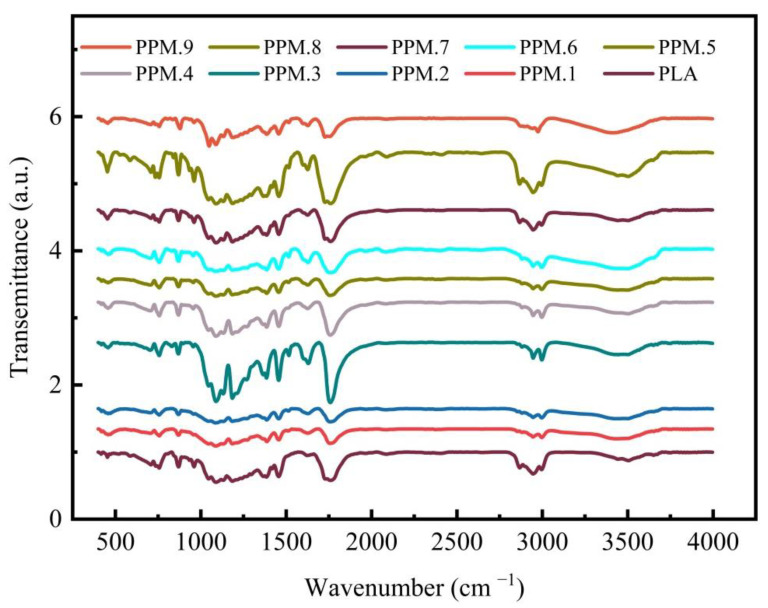
FTIR spectra of pure PLA and PLA films loaded with different amounts of the phlridzin/MCM-41 assembly.

As shown in Figure S2, the infrared spectrum of MCM-41 loaded with phloridzin contains the characteristic peaks of the two raw materials, and the results show that phlorizin is fully loaded on MCM-41. And as shown in Figure 5 and Figure S3, a relatively strong absorption peak appears at 1750 cm^−1^, corresponding to the carbon-based characteristic absorption peak of PLA. Peaks at 1457 and 1514cm^−1^ correspond to the benzene ring in phloridzin. The peak at 1620 cm^−1^ corresponds to the carbonyl group in phloridzin, and the peak at 1184 cm^−1^ corresponds to the characteristic CO absorption peak of PLA. With the increase of added phloridzin, the peak intensity also increases. Compared with the pure PLA film, the infrared spectrum of the film with the added assembly shows two new absorption peaks at 1475 and 1514 cm^−1^, corresponding to the phenyl in the phloridzin molecule. The peak at 455 cm^−1^ corresponds to the bending vibration of the silicon-oxygen bond in the MCM-41 structure. The peak intensity increases with assembly, which also demonstrates the presence of assembly in the film. Compared with the pure PLA film, the film with the added assembly only shows the characteristic peak of the assembly, with no other absorption peaks appearing. This result shows that after the electrospinning film formation, not only are the phloridzin and MCM-41 stable without decomposition, but there is also no chemical reaction occurring between the components of the controlled release film.

### 3.6. Research on the Oxidation Resistance of the Film

**Figure 6:**
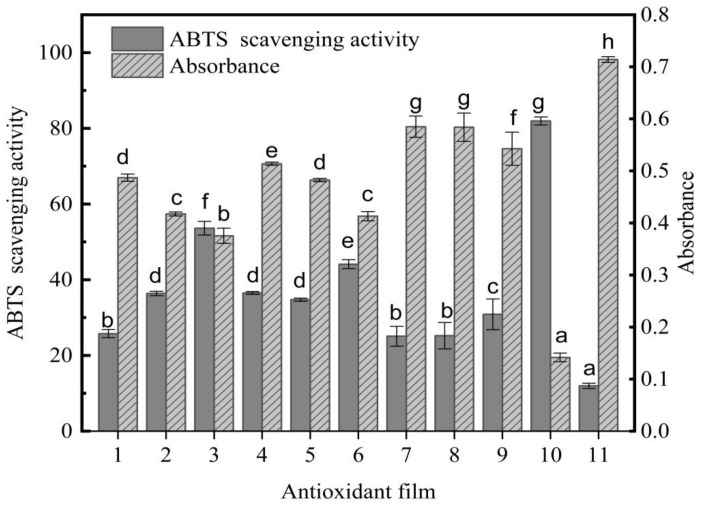
The antioxidant capacity of the anti-oxidative film, expressed as the percentage of ABTS radical inhibition and the absorbance at 734 nm. Different letters indicate significant differences between films (*p* < 0.05).

The scavenging rates of ABTS free radicals by antioxidative films, phloridzin, and pure PLA films assembled with different components are shown in Figure 6. On the *x*-axis, 1 to 9 are active films with different components, 10 is phloridzin, and 11 is pure PLA film.

The ABTS free-radical scavenging rate of the PLA antioxidant film is shown in Figure 6. As can be seen, the scavenging rate of ABTS free radicals continued to increase with the rise of the antioxidant phloridzin content. The ratio of phloridzin to molecular sieve in sample 3 achieved the highest ABTS free-radical scavenging rate of 53.61%. In a previous study, Fematt-Flores et al. [43] prepared an antioxidant film to inhibit ABTS free radicals (32.64%), showing that the antioxidant film in this study would perform well. Moreover, when the ratio of phloridzin to molecular sieve was equal, the greater the phloridzin content, the higher the ABTS free-radical scavenging rate of the antioxidant film, and the better the antioxidant performance. Phloridzin has strong antioxidant activity, and its own ABTS free-radical scavenging rate is 81.95%. When phloridzin in the film is dissolved in a solvent, phloridzin can scavenge ABTS free radicals due to its strong antioxidant properties. In addition, as the phloridzin concentration increases, the scavenging rate of ABTS free radicals also gradually increases.

### 3.7. Research on the Antibacterial Properties of the Film

**Figure 7:**
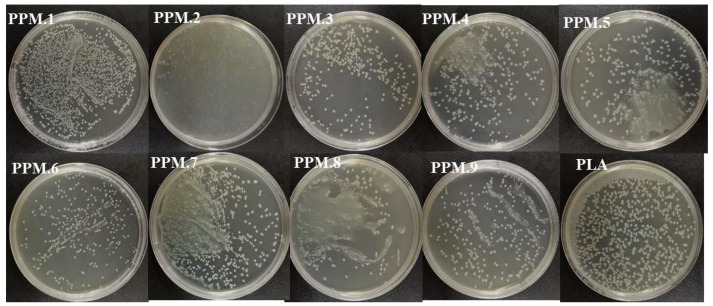
Antibacterial effect of antioxidant films containing assemblies of different amounts.

**Figure 8:**
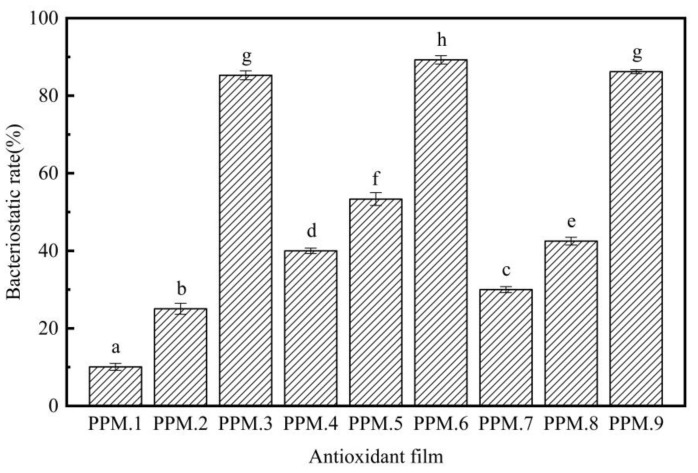
Antibacterial effect of MCM-41/phloridzin/PLA antioxidant films containing different component assemblies against *Escherichia coli* (*p* < 0.05).

The analysis of the inhibitory effect of the antioxidant film on *Escherichia coli* by the plate counting method are shown in Figure 7, Figure 8 and Table 7. 

As shown in Figure 7, Figure 8 and Table 7, compared with the pure PLA membrane in the control group, the antibacterial effect of the fiber membrane on *E. coli* gradually increased with the increased content of phloridzin in the assembly samples. According to Figure 7, the pure PLA film had almost no antibacterial properties. Thus, pure PLA can be used as the blank control group. The film with the highest antibacterial rate and the best antibacterial performance was the one that contained the assembly with the highest amount of phloridzin. The rates were 85.27 ± 1.72%, 89.25 ± 1.07%, and 86.20 ± 0.50%. When the ratio of phloridzin to molecular sieve was 3:4, the antibacterial rate of the film was high (89.25 ± 1.07%), and the antibacterial performance was excellent.

### 3.8. Mechanical Properties of the Film

The analysis of the mechanical properties of the antioxidant-active film is shown in Table 8.

As can be seen, the mechanical properties of the film were affected by the combined effect of mesoporous materials and active substances. The addition of a large amount of active material and mesoporous material assembly to the film damages the uniformity of the film, so the film eventually exhibits a decrease in tensile properties. With the increase of mesoporous materials and active substances in the assembly, the mechanical properties of the film gradually decrease. The mechanical properties become better with the addition of the mesoporous molecular sieve.

### 3.9. Water Vapor Transmission Rate of the Film

The water vapor transmission rates of the antioxidant films of each component are shown in Table 9 (*p* < 0.05).

According to Table 9, the pure PLA film had the highest water vapor transmission (WVP) rate, while the PLA film containing other components had a lower WVP rate. This result is because the hydrophilic group of phloridzin enhances the hydrophilicity of the film, thereby increasing the WVP rate of the film. However, the controlled release film containing the assembly had a low WVP rate. Although phloridzin is hydrophilic, the MCM-41 mesoporous molecular sieve and PLA are hydrophobic. This component of MCM-41/PLA effectively prevents the transmission of water vapor. Therefore, even the phloridzin in the antioxidant film was not sufficient to significantly increase the WVP rate.

### 3.10. Application of Film in Strawberry Preservation

#### 3.10.1. Sensory Evaluation

**Figure 9:**
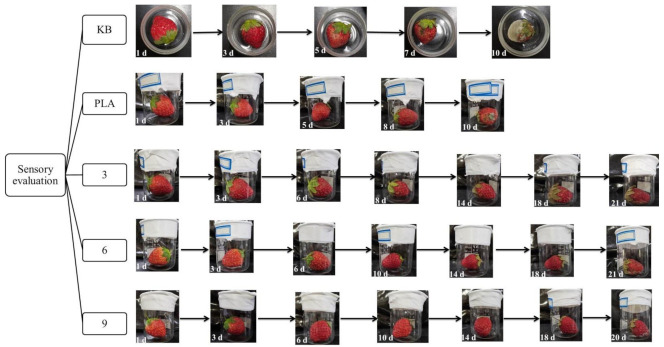
The fresh-keeping effect of blank no film, pure PLA film, and antioxidant food packaging film.

Due to microbial action, oxidation, respiration, enzyme action, and mechanical damage over time, the internal nutrients of stored strawberries decompose and change, resulting in rot. The color, shape, and taste of fruits and vegetables will change, losing their nutritional value. The anti-oxidation films of phloridzin with different component contents were used as the sample to determine the preservation effect. The results of the freshness test are shown in Figure 9. As can be seen, the freshness preservation effect of the strawberry without any packaging film is extremely poor. After 5 days at room temperature, a part of the strawberry surface began to mold, and after 10 days at room temperature, the strawberry was completely moldy.

The fresh-keeping effect of the pure PLA film is unsatisfactory. After 7 days at room temperature, moldy spots appeared on the surface of the strawberry, indicating that it was no longer suitable for consumption. After 10 days at room temperature, the strawberry was extensively moldy. The active food packaging film in this study was found to have a good fresh-keeping effect. After being placed at room temperature for 10 days, the strawberry remained free of mildew and rot. When the mass ratios of phloridzin to molecular sieve were 2:1 and 4:3, the freshness preservation effect was the best, with moldy spots appearing after 20 days. Compared with the blank control group, other ratios of active packaging films had better fresh-keeping effects, and mildew spots only appeared after 15 days of storage. In the literature [44], a chitosan/corn starch/cinnamaldehyde film for strawberry fresh-keeping was prepared, and the active food packaging film was able to extend the shelf-life of strawberries to about 11 days. It can be seen by comparison that the antioxidant film in this study has a very good effect on strawberry preservation.

**Figure 10:**
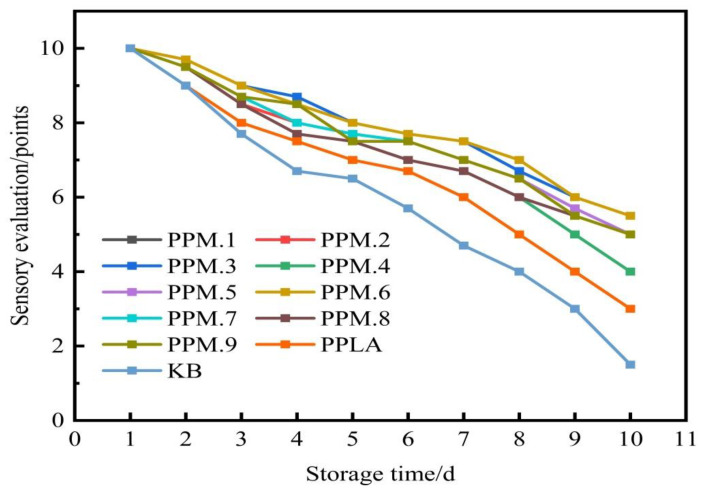
The effect of the addition of the assembly on the sensory evaluation of strawberries.

Taking sensory evaluation as the standard, the number of days when the strawberry color reaches the same depth and mold spots are shown in Figure 10. It can be observed from Figure 10 that the surface of the strawberry under the sealing condition of the active food packaging film was still relatively smooth and red after 10 days. After 7 days, moldy spots appeared on the surface of the strawberries sealed with the pure PLA film, and the color of the strawberries became darker. Strawberries stored under the condition of no packaging film quickly become moldy, and the freshness preservation effect was poor.

The experiment shows that the antioxidant film has a good preservation effect on strawberries. The reason being that the phloridzin in the film has good antioxidant properties. After the film has been formed with the electrostatic spinning technology, the antioxidant properties are not destroyed, and the resulting film has a good fresh-keeping effect. This means that the film can be used for the preservation of fruits and vegetables with a great effect.

#### 3.10.2. Weightlessness Rate

**Figure 11:**
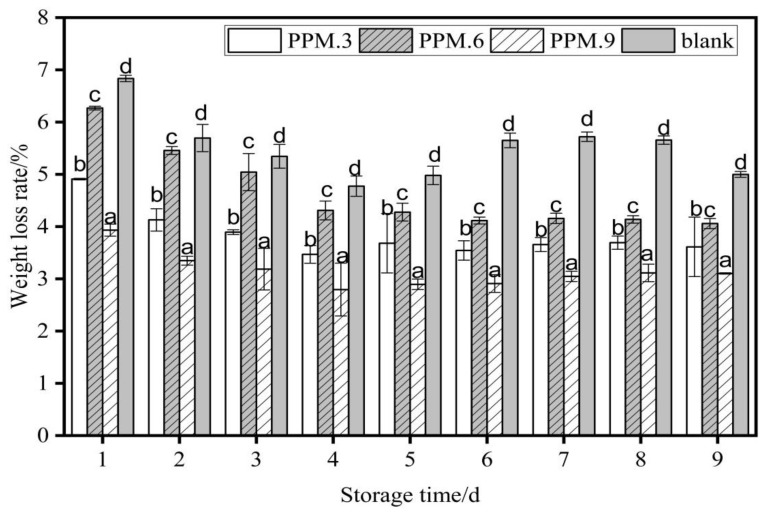
The effect of antioxidant film of each component content on the weight loss rate of strawberry (*p* < 0.05). Data are presented as mean ± standard deviation, and different letters (a–d) within the columns show the significant differences (*p* < 0.05), where is the lowest value.

The effects of samples No. 3, 6, 9, and the blank components in the antioxidant film on the weight loss rate of strawberries (*p* < 0.05) are shown in Figure 11.

As shown in Figure 11, the weight loss rate increased with the number of storage days. The weight loss rate of strawberries in the blank control group was always higher than that in the film-sealed group. The weight loss rate during the same storage time was highest for the blank control, then pure PLA film, and finally active food packaging film had the lowest loss rate. The film plays a role in antioxidant activity, isolates the external environment, and achieves the effect of preservation. After 10 days of storage, when the added amount of phloridzin was 0.2 g, the weight loss rate of strawberries reached a minimum of 3.10 ± 0.01%, and under the same conditions, the weight loss rate of strawberries in the control group was 5.00 ± 0.05%. The weight loss rate of strawberries under the action of other films was higher than 3.10 ± 0.01%. Therefore, when the added amount of phloridzin was 0.2 g, the fresh-keeping effect of the packaging film for samples 3, 6, and 9 was relatively better.

## 4. Conclusions

Phloridzin was loaded on to an MCM-41 mesoporous material, and a novel antioxidant-loaded active packaging film was developed using the electrospinning method. In optimizing the preparation process, the highest loading rate obtained for an assembly was 30.54%. Based on the ABTS free-radical-scavenging experiment, the antioxidant properties of the film before and after loading phloridzin were studied. The results show that the antioxidant effect of film sample 3 (PPM.3, loading rate 30.54%) was 41.64% higher than that of pure PLA film. In terms of antibacterial activity, when the added amount of phloridzin is the highest, and the mass ratio to MCM-41 is 4:3, the highest bacteriostatic rate is 89.25 ± 1.07 ^h^%. 

To evaluate the antioxidant effect of phloridzin on food packaging film, the novel packaging film was used in an experiment to assess the freshness of strawberries. The packaging film was found to extend the shelf-life of strawberries to about 20 days. The results showed that the adsorption of phloridzin on MCM-41 had a significant effect on the preservation of strawberries. Therefore, MCM-41-loaded phloridzin active food packaging film was successfully prepared in this study, and a new perspective for the research of phloridzin in food antioxidants was provided. In follow-up studies, the mechanical properties of the base PLA material in the film will be modified to improve the mechanical properties of the active packaging film and be applied to food packaging.

## Figures and Tables

**Table 1:** Experimental instruments and equipment.

Instrument Name	Instrument Model	Manufacturing Company
Electrospinning machine	YFSP-T	Tianjin Yunfan Technology Co., Ltd. (Tianjin, China)
Electric heating blast drying oven	GZX-9240MBE	Shanghai Boxun Industrial Co., Ltd. Medical equipment (Shanghai, China)
Electronic balance	FA2004	Shanghai Yueping Scientific Instrument Co., Ltd. (Shanghai, China)
High-power magnetic stirrer	DJ-I	Changzhou Aohua Instrument Co., Ltd. (Changzhou, China)
Sterile disposable syringe	FF	Jiangsu Huada Medical Instrument Co., Ltd. (Jiangsu, China)
Deyisheng Ultrasonic Cleaner	040S	Shenzhen Huace Technology Co., Ltd. (Shenzhen, China)
Air humidifier	HU4706	Philips (Shanghai, China) Investment Co., Ltd.
Scanning Electron Microscope	NOVANANOSEM-450	FEI Company, (Hillsboro, OR, USA)

**Table 2:** Assembling preparation process conditions in orthogonal experiment.

	MCM-41	Antioxidant	Stirring Time
1	Level 1 (0.1 g)	Level 1 (0.0.5 g)	Level 1 (8 h)
2	Level 1	Level 2 (0.1 g	Level 2 (12 h)
3	Level 1	Level 3 (0.2 g)	Level 3 (16 h)
4	Level 2 (0.15 g)	Level 1	Level 2
5	Level 2	Level 2	Level 3
6	Level 2	Level 3	Level 1
7	Level 3 (0.2 g)	Level 1	Level 3
8	Level 3	Level 2	Level 1
9	Level 3	Level 3	Level 2

**Table 3:** Sensory evaluation of strawberries.

Score	Appearance	Color	Smell
8–10 points	good appearance, fruit color	bright red	fruit fragrance
6–8 points	good appearance, partial dehydration of the fruit	slightly lighter color	slight fruit fragrance
4–6 points	heavier water loss, wilting,	dark red fruit	slightly sour
2–4 points	partially rotten, moldy	fruit turns black	slightly musty
0–2 points	all rotten, moldy	the fruit turns black and rotten	all with a rotten smell

**Table 4:** The loading rate of phloridzin in phloridzin/MCM-41 assembly.

Sample Serial Number	PPM.1	PPM.2	PPM.3	PPM.4	PPM.5	PPM.6	PPM.7	PPM.8	PPM.9
Loading rate of phloridzin (%)	14.99	22.69	30.54	11.29	21.86	26.31	16.68	14.92	24.55

**Table 5:** Loading rate at every level and between levels for each factor.

	Factor	I	II	III
Level				
Loading rate	1	68.22	42.96	56.22
	2	59.46	59.47	58.53
	3	56.15	81.4	69.08
	Extreme difference (R)	12.07	38.44	12.86

**Table 6:** Specific area and pore volume of MCM-41 before and after loading.

Phloridzin/MCM-41	S (m^2^·g^−1^)	V (cm^3^·g^−1^)
MCM-41	890.740	0.961
PPM.1	282.920	0.379
PPM.2	92.693	0.157
PPM.3	59.504	0.133
PPM.4	734.720	0.767
PPM.5	98.438	0.230
PPM.6	34.598	0.048
PPM.7	466.120	0.532
PPM.8	110.950	0.138
PPM.9	34.448	0.059

**Table 7:** Antibacterial effect of MCM-41/phloridzin/PLA antioxidant films containing different component assemblies against *Escherichia coli* (*p* < 0.05).

Different R of Membranes	1	2	3	4	5	6	7	8	9
Inhibition rate (%)	10.07 ± 0.89 ^a^	25.05 ± 1.42 ^b^	85.27 ± 1.72 ^g^	40.00 ± 0.70 ^d^	54.94 ± 1.66 ^f^	89.25 ± 1.07 ^h^	30.02 ± 0.76 ^c^	42.50 ± 1.00 ^e^	86.20 ± 0.50 ^g^

Data are presented as mean ± standard deviation, and different letters (a–h) within the columns show the significant differences (*p* < 0.05), where is the lowest value.

**Table 8:** Mechanical properties of the pure PLA film and the film added with the assembling (*p* < 0.05).

	Modulus of Elasticity	Elongation at Break	Tensile Stress at Break	Tensile Strength
	MPa	%	MPa	MPa
PLA	0.70 ± 0.10 ^a,b^	13.24 ± 0.85 ^b^	0.10 ± 0.06 ^a,b^	0.14 ± 0.04 ^a^
1	7.08 ± 0.93 ^d^	13.97 ± 0.85 ^b^	0.2 ± 0.10 ^b^	0.14 ± 0.02 ^a^
2	17.07 ± 0.88 ^e^	10.3 ± 0.54 ^b^	0.11 ± 0.06 ^a,b^	0.13 ± 0.05 ^a^
4	2.44 ± 0.84 ^c^	10.1 ± 0.70 ^a^	0.04 ± 0.02 ^a,b^	0.12 ± 0.04 ^a^
5	1.78 ± 0.25 ^b,c^	10.22 ± 1.15 ^a^	0.11 ± 0.03 ^a,b^	0.14 ± 0.04 ^a^
6	0.54 ± 0.11 ^a^	9.82 ± 0.33 ^a^	0.12 ± 0.04 ^a,b^	0.07 ± 0.07 ^a^

Results are reported as the mean ± standard deviation. Different lowercase letter within each column indicates significant statistical differences (*p* < 0.05) between films. Data are presented as mean ± standard deviation, and different letters (a–c) within the columns show the significant differences (*p* < 0. 05), where a is the lowest value.

**Table 9:** The water vapor transmission rate of the antioxidant films of each component (*p* < 0.05).

Antioxidant Films	PPM.1	PPM.2	PPM.3	PPM.4	PPM.5	PPM.6	PPM.7	PPM.8	PPM.9	PLA
WVP(g·mm/m^2^·h·KPa)	0.72 ± 0.03 ^f^	0.75 ± 0.08 ^g^	0.82 ± 0.26 ^h^	0.4 ± 0.05 ^c^	0.41 ± 0.12 ^d^	0.64 ± 0.07 ^e^	0.33 ± 0.07 ^a^	0.39 ± 0.09 ^b^	0.41 ± 0.07 ^d^	1.14 ± 0.07 ^i^

Data are presented as mean ± standard deviation, and different letters (a–i) within the columns show the significant differences (*p* < 0. 05), where is the lowest value.

## Data Availability

The data presented in this study are available on request from the corresponding author.

## Supplementary Materials

The following are available online at https://www.mdpi.com/2079-4991/12/7/1229/s1, Figure S1: Nanofibers and size distribution of PLA and samples 2, 6 and 9, Figure S2: Infrared images of MCM-41, phlorizin and assembly sample 1, Figure S3: Infrared spectrum of pure PLA as thin film sample 1.
