# Correction: Xie et al. Preparation and Characterization of New Electrospun Poly(Lactic Acid) Nanofiber Antioxidative Active Packaging Films Containing MCM-41 Mesoporous Molecular Sieve Loaded with Phloridzin and Their Application in Strawberry Packaging. *Nanomaterials* 2022, *12*, 1229

**DOI:** 10.3390/nano12172932

**Published:** 2022-08-25

**Authors:** Yuan Xie, Guiguang Cheng, Zhoushan Wu, Shang Shi, Jinghao Zhao, Lin Jiang, Dengbang Jiang, Mingwei Yuan, Yudan Wang, Minglong Yuan

**Affiliations:** 1 School of Chemistry and Environment, National and Local Joint Engineering Research Center for Green Preparation Technology of Biobased Materials, Yunnan Minzu University, Kunming 650500, China; 2 Faculty of Food Science and Engineering, Kunming University of Science and Technology, Kunming 650500, China; 3 Key Laboratory of Chemistry in Ethnic Medicinal Resources, State Ethnic Affairs Commission & Ministry of Education, Yunnan Minzu University, Kunming 650500, China

## Error in Figure

In the original publication, there was a mistake in Figure 7, as published in [1]. Upon building up the combined image of PPM.7 and PPM.8, the authors misplaced the panel corresponding to the previously mentioned samples.

The authors apologize for any inconvenience caused and state that the scientific conclusions are unaffected. This correction was approved by the Academic Editor. The original publication has also been updated.

Original image (incorrect one):


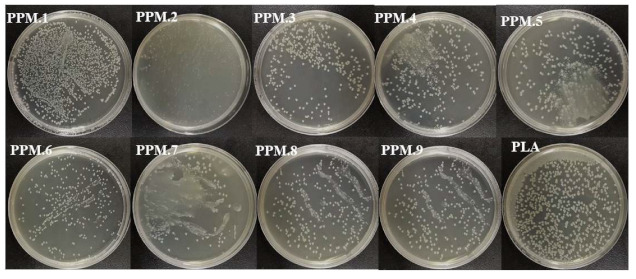


Corrected Figure 7:

**Figure 7:**
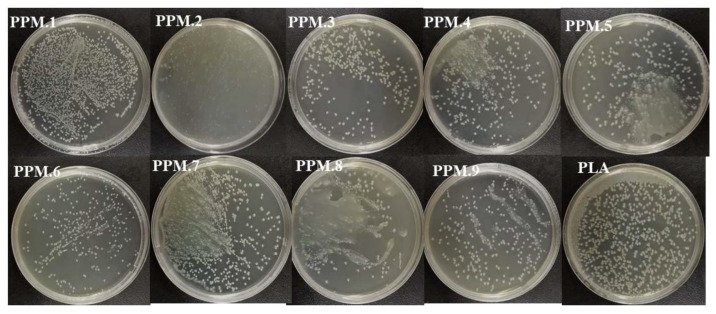
Antibacterial effect of antioxidant films containing assemblies of different amounts.
