# Supplementary material for: Preparation and Characterization of New Electrospun Poly(lactic acid) Nanofiber Antioxidative Active Packaging Films Containing MCM-41 Mesoporous Molecular Sieve Loaded with Phloridzin and Their Application in Strawberry Packaging
Source: Nanomaterials (Basel). 2022 Apr 6;12(7):1229. doi: 10.3390/nano12071229 (PMC9000760; doi:10.3390/nano12071229)
Supplement: Supplementary file 1 [file nanomaterials-12-01229-s001.zip › nanomaterials-1640470-supplementary.pdf]

nanomaterials

Supplementary Materials

Preparation and Characterization of New Electrospun Poly(lactic acid) Nanofiber Antioxidative Active Packaging Films Containing MCM-41 Mesoporous Molecular Sieve Loaded with Phloridzin and their Application in Strawberry Packaging

Yuan Xie [1], Guiguang Cheng [2], Zhoushan Wu [3], Shang Shi [3], Jinghao Zhao [3], Lin Jiang [1], Dengbang Jiang [1], Mingwei Yuan [1], Yudan Wang [1,3,*] and Minglong Yuan [1,*]

[1] School of Chemistry and Environment, National and Local Joint Engineering Research Center for Green Preparation Technology of Biobased Materials, Yunnan Minzu University, Kunming 650500, China; yuan18787028972@sina.com (Y.X.); ggcheng@kust.edu.cn (G.C.); jianglin@ymu.edu.cn (L.J.); 041814@ymu.edu.cn (D.J.); 041808@ymu.edu.cn (M.Y.)
[2] Faculty of Food Science and Engineering, Kunming University of Science and Technology, Kunming 650500, China
[3] Key Laboratory of Chemistry in Ethnic Medicinal Resources, State Ethnic Affairs Commission & Ministry of Education, Yunnan Minzu University, Kunming 650500, China; 21227038010037@ymu.edu.cn (Z.W.); 15807220798@sina.cn (S.S.); jinghaozhao1019@sina.com (J.Z.)
* Correspondence: wangyudan@ymu.edu.cn (Y.W.); yml@ynni.edu.cn (M.Y.)

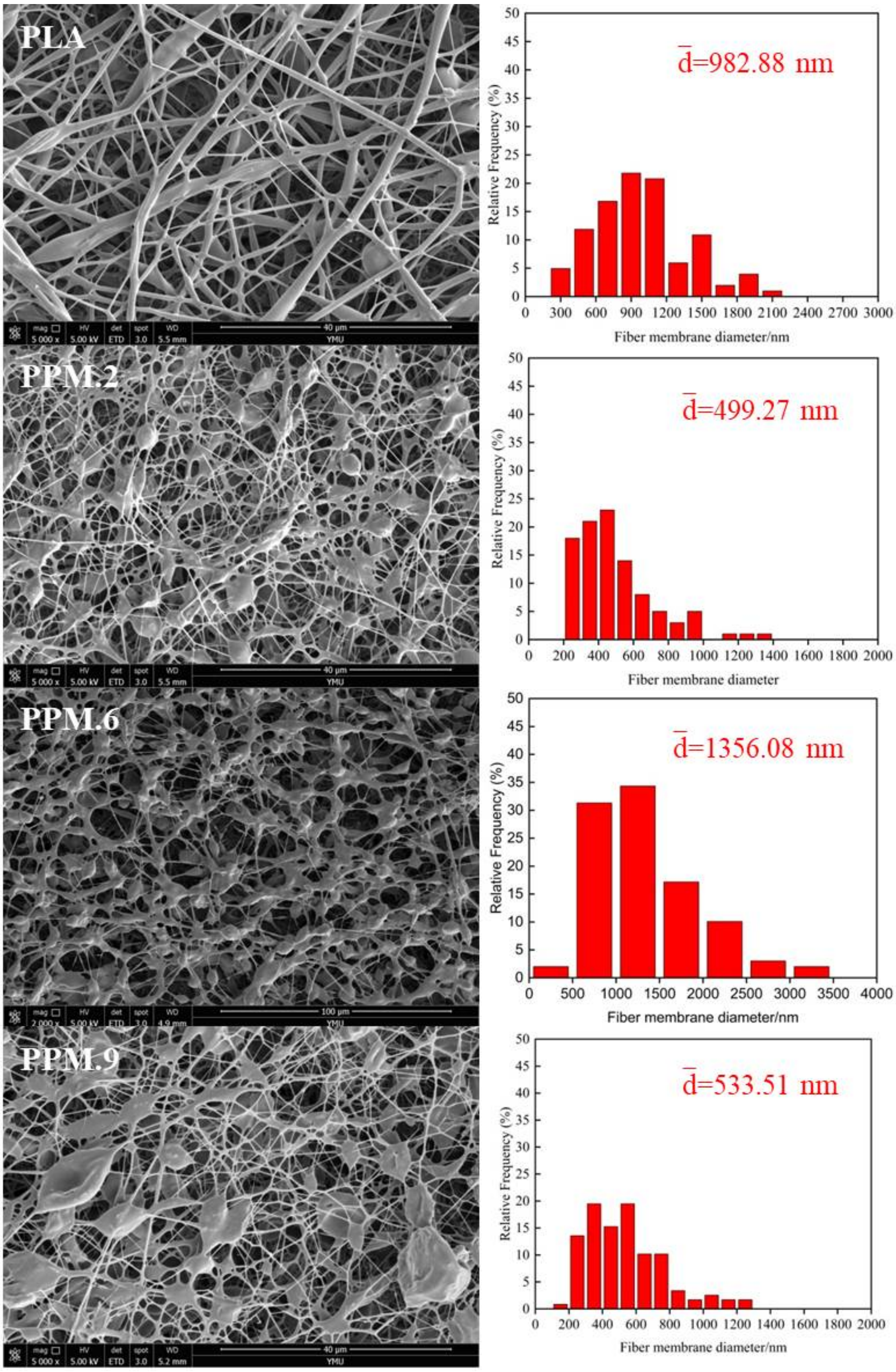

Figure S1. Nanofibers and size distribution of PLA and samples 2, 6 and 9.

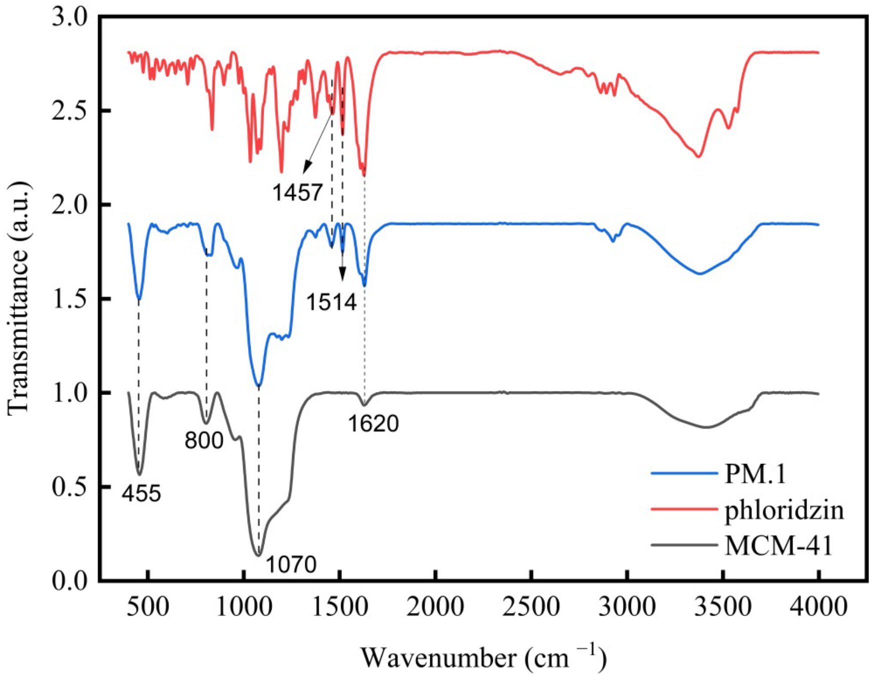

Figure S2. Infrared images of MCM−41, phlorizin and assembly sample 1.

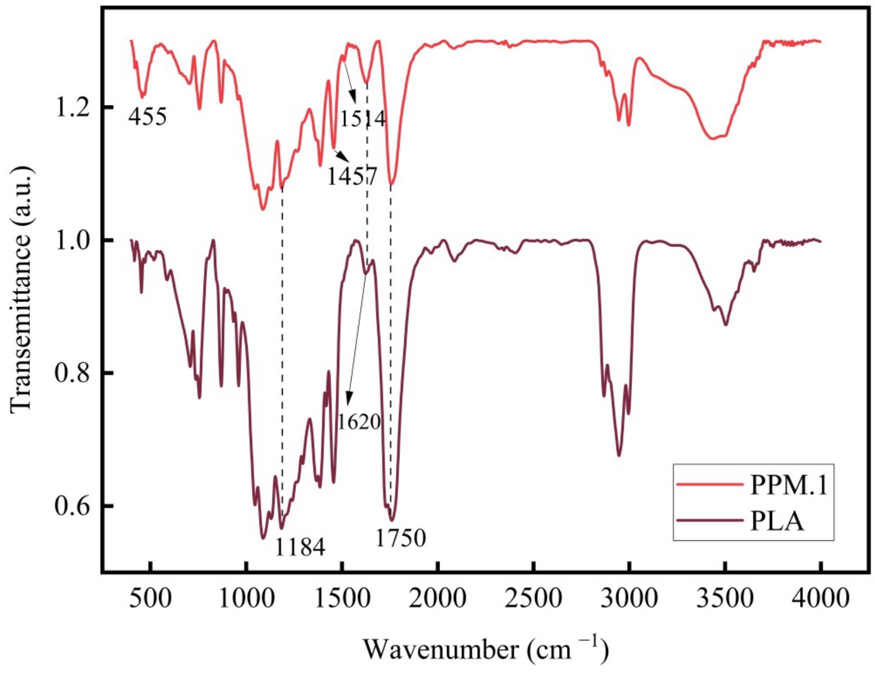

Figure S3. Infrared spectrum of pure PLA as thin film sample 1.
